# 

**Published:** 1855-10

**Authors:** 


PUBLISHED MONTHLY, AT $3 PER ANNUM IN ADVANCE.

### ATLANTA MEDICAL AND SURGICAL JOURNAL.

EDITED BY
**JOSEPH P. LOGAN, M. D.,**
Professor of Physiology and General Pathology,
AND
**W. F. WESTMORELAND, M. D.,**
Professor of the Principles and Practice of Surgery.

SMITHSONIAN INSTITUTION

Pax et scientia, sed veritas sine timore.

Vol. I. OCTOBER, 1855. No. 2.

ATLANTA, GEORGIA:

PRINTED BY C. R. HANLEITER AND CO.

1855.

☞ Each Number contains sixty-four pages. Postage.—Under 500 miles, 15 cents a year; over 500 miles, 30 cents a year—to be pre-paid quarterly. If not pre-paid, double the above rates.

